# Considerations on the development of surgical techniques for the treatment of onychocryptosis^☆^^☆☆^

**DOI:** 10.1016/j.abd.2021.06.002

**Published:** 2021-07-23

**Authors:** Anna Carolina Miola, Giovana Piteri Alcantara, Luciane Donida Bartoli Miot, Helio Amante Miot

**Affiliations:** Department of Dermatology, Faculdade de Medicina de Botucatu, Universidade Estadual Paulista, Botucatu, SP, Brazil

Dear Editor,

As onychocryptosis is a frequent demand in dermatological assistance, and its surgical management requires both specific training and indication criteria, we read with interest the article by Ma,^1^ which aimed to describe a new surgical approach for onychocryptosis.

Currently, there is no consensus, nor a body of evidence on the specific differences of the several surgical techniques for onychocryptosis, or on their comparison in terms of effectiveness, morbidity, infection, cost-effectiveness and technical difficulty. Therefore, the development of new methods is of scientific relevance and should be critically appreciated considering the described surgeries, especially regarding the technical differences and recurrence rates after 12 months.

Despite the interesting results presented by Dr. Ma, the proposed surgical technique sequence is very similar to the classic matricectomy described by Winograd (1929),^2^ which has undergone several adaptations over the years.^3^, ^4^ Moreover, although low, there is an expected recurrence rate of approximately 6% in virtually all studies that used the Winograd method or its variants.^4^ As this is a similar surgical approach, the result shown by Ma, who found no recurrence in 67 surgeries (with a follow-up of 6 to 12 months), may not represent a difference in relation to the expected rate of 6% (p = 0.119 – Fisher Exact test) due to modest sample size. However, it can also be due to the small percentage of cases with grade I onychocryptosis, which usually do not show recurrence and whose frequency was not discriminated by the author.

Table 1 depicts the main technical characteristics of the Winograd method and its main variants, its recurrence rates, in addition to chemical matricectomy with 88% phenol and 80% trichloroacetic acid, for comparison.^5^

**Table 1:** Characteristics of the main surgical techniques described for onychocryptosis.

Publication	Technique	n	Recurrence rate
Winograd AM. J Am Podiatr Med Assoc 2007; 97:274-7.	Incision of the eponychium	10	Zero in 6 months
	Removal of the nail plate to the matrix		
	Matrix curettage, dressing		
Uygur E, et al. Int J Surg 2016; 34:1-5.	Winograd method	128	14% in 6 months
	Posterior suture of the surgical wound on the nail plate		
Acar E. J Foot Ankle Surg 2017; 56:474-7	Winograd method	102	Zero in 12 months
	Electrocoagulation of the matrix		
Karaca N. et al. Ann Fam Med 2012; 10: 556-9	Partial excision of the proximolateral matrix and phenolization of the matrix	348	0.3% in 24 months
Aksoy, et al. Dermatol Surg 2009; 35:462-8.	Transposition flap of proximal nail fold with partial matricectomy	52	3.9% in 12 months
Osan F, et al. Dermatol Surg 2014; 40:1132-9.	Partial matricectomy with curettage (group 1) vs. Partial matricectomy with electrocauterization (group 2)	92 (group 1) *vs*57 (group 2)	2% in 10 meses (group 1) *vs.* Zero in 10 months (group 2)
Dąbrowski M, et al. Ann Med Surg 2020; 56:152-160.	Wedge-shaped excision of tissue lateral to the nail plate	54	1.8% in 11 months
	Preservation of the nail matrix		
Kimata, et al. Plast Reconstr Surg 1995; 95:719-24.	Partial avulsion of the lateral nail plate	537	1% in 6 months
	Chemical matricectomy with 88% phenol		
Barreiros H, et al. An Bras *Dermatol* 2013; 88:889-93.	Partial avulsion of the nail plate and lateral nail matrix	197	1.5% in 12 months
	Chemical matricectomy with 80% trichloroacetic acid		
Muriel-Sanchez JM, et al. J Clin Med 2020; 9:845.	Avulsion of the lateral nail plate to the eponychium and curettage of the nail matrix and nail bed (group 1) vs. Nail plate avulsion to the eponychium and 88% phenolization of the nail matrix (group 2)	76 (group 1) *vs*. 36 (group 2)	1.52% in 6 meses (group 1) *vs.* 2.8% in 6 months (group 2)
Montesi S, et al. Dermatology 2019; 235:323-6.	88% phenolization of the nail matrix for 4 minutes	622	1.1% in 12 months
Terzi E, et al. Dermatol Surg 2017; 43: 728-33.	Avulsion of the lateral nail plate to the eponychium and chemical matricectomy with 90% dichloroacetic acid	58	3.3% in 12 months

Surgical techniques for the treatment of onychocryptosis require careful systematization of the operative sequences and approach to the matrix, as well as the precise indication according to tissue hyperplasia, nail plate situation and pyogenic granuloma. Only the comparative analysis of the performance of the techniques, stratified according to the indications, can lead to criticism, aiming to maximize the performance of the procedures.

Due to the peculiar anatomy of the nail apparatus, surgical approaches to onychosis require specialized training by the dermatologist. However, despite the high prevalence of onychocryptosis and impact on quality of life, there is a lack of well-conducted comparative clinical trials that favor the personalization of indications. Moreover, it is crucial to review the previously described surgical techniques, both for their historical and scientific value, when one proposes the standardization of a new surgical technique.

## Financial support

None declared.

## Authors’ contributions

Anna Carolina Miola: Approval of the final version of the manuscript; drafting and editing of the manuscript; critical review of the literature; critical review of the manuscript.

Giovana Piteri Alcantara: Drafting and editing of the manuscript; critical review of the manuscript.

Luciane Donida Bartoli Miot: Drafting and editing of the manuscript; critical review of the manuscript.

Hélio Amante Miot: Approval of the final version of the manuscript; drafting and editing of the manuscript; critical review of the literature; critical review of the manuscript.

## Conflicts of interest

None declared.
